# A case of disseminated carcinomatosis of the bone marrow originating from gastric cancer 3 years after intraperitoneal chemotherapy against peritoneal carcinomatosis

**DOI:** 10.1186/s12957-016-0851-3

**Published:** 2016-04-14

**Authors:** Takayuki Okuno, Hironori Yamaguchi, Joji Kitayama, Hironori Ishigami, Takeshi Nishikawa, Junichiro Tanaka, Toshiaki Tanaka, Tomomichi Kiyomatsu, Keisuke Hata, Hiroaki Nozawa, Kazushige Kawai, Shinsuke Kazama, Soichiro Ishihara, Eiji Sunami, Toshiaki Watanabe

**Affiliations:** Department of Surgical Oncology, The University of Tokyo, 7-3-1 Bunkyo-ku, Tokyo, 113-8655 Japan; Department of Chemotherapy, The University of Tokyo, Tokyo, Japan

**Keywords:** Gastric cancer, Disseminated carcinomatosis of the bone marrow, Intraperitoneal chemotherapy

## Abstract

**Background:**

Clinical studies of intraperitoneal chemotherapy with paclitaxel in patients of gastric cancer with peritoneal carcinomatosis is well tolerated and effective, and rare cases of metastasis and recurrence have experienced during the treatment. Disseminated carcinomatosis of the bone marrow is highly rare in gastric cancer and associated with a poor prognosis.

**Case presentation:**

A 59-year-old woman of gastric cancer with peritoneal carcinomatosis received five courses of chemotherapy with intraperitoneal administration of paclitaxel, and laparoscopy showed disappearance of the peritoneal carcinomatosis. She subsequently underwent total gastrectomy, and the histopathological findings showed a complete response to the chemotherapy. Postoperatively, chemotherapy with intraperitoneal administration of paclitaxel was continued for 30 months, without apparent recurrence. However, the gastric cancer recurred as disseminated carcinomatosis of the bone marrow with disseminated intravascular coagulation, and we hence changed the chemotherapy regimen to weekly irinotecan. Remission was achieved, and she did not experience any major symptoms; however, she died 6 months after the diagnosis of disseminated carcinomatosis of the bone marrow.

**Conclusions:**

Since intraperitoneal paclitaxel administration can strongly suppress peritoneal carcinomatosis of gastric cancer, careful attention should be paid not only to peritoneal recurrence but also for rare site metastases, such as bone marrow metastases.

## Background

Peritoneal carcinomatosis is the most frequent mode of metastasis and recurrence in patients with gastric cancer. Clinical studies investigating the effects of intraperitoneal chemotherapy with paclitaxel (PTX) performed by our group on such patients have shown that the treatment is well tolerated and effective [1–3]. Moreover, we have experienced rare cases of metastasis and recurrence recently, such as leptomeningeal carcinomatosis [4] after long-term control of peritoneal metastasis. Particularly, bone marrow metastasis is highly rare in gastric cancer [5], and disseminated carcinomatosis of the bone marrow (DCBM) is known to be closely associated with disseminated intravascular coagulation (DIC) and a poor prognosis [5, 6]. Herein, we present a rare case of DCBM originating from gastric cancer and developing 3 years after intraperitoneal chemotherapy with PTX against peritoneal carcinomatosis.

## Case presentation

**Fig. 1:**
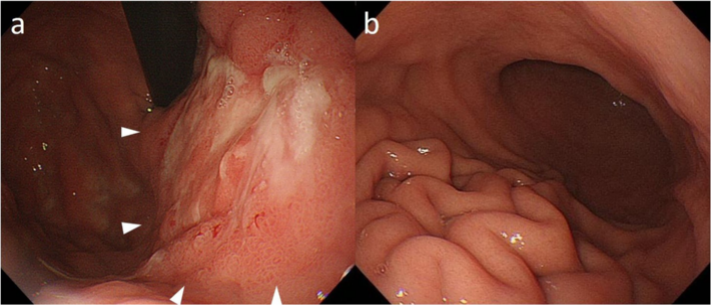
Upper gastrointestinal endoscopic studies of the patient. **a** Borrmann type IV gastric cancer with ulceration at the upper part of the stomach was revealed. **b** Giant folds of the greater curvature and poor extension of the wall were shown at the middle part of the stomach

**Fig. 2:**
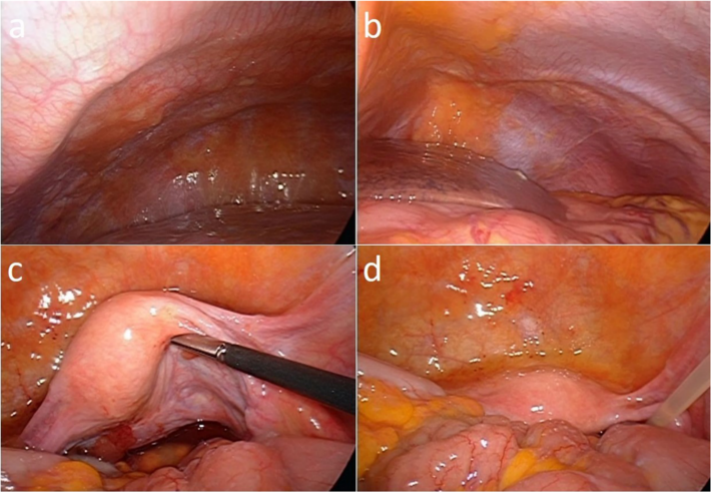
Laparoscopic studies of the patient. Repeat laparoscopy is showing a disappearance of the peritoneal carcinomatosis. **a** Right upper quadrant of the abdomen. **b** Left upper quadrant of the abdomen. **c**, **d** Lower part of the abdomen

**Fig. 3:**
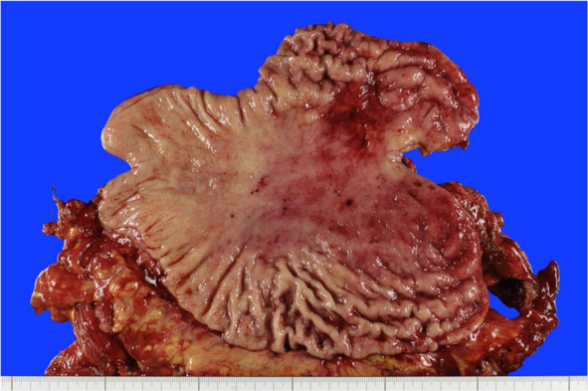
Resected specimen of the patient. Pathological examination revealed no viable cancer cells remaining in the specimen

A 59-year-old woman was referred to our department for intraperitoneal chemotherapy for gastric cancer with peritoneal carcinomatosis. She had already received two courses of chemotherapy with S-1 plus cisplatin in the previous hospital. Upper intestinal endoscopy showed Borrmann type IV gastric cancer with ulceration at the upper part of the stomach (Fig. 1a, b). Pathological diagnosis was poorly differentiated tubular adenocarcinoma with the signet ring cell carcinoma. Staging laparoscopy showed a thickened omentum due to carcinomatosis (“omental cake”) and numerous disseminated nodules in the whole abdominal cavity. An intraperitoneal access port was implanted to allow for chemotherapy administration. After the staging laparoscopy, she received five courses of chemotherapy with intraperitoneal administration of PTX. At first, two courses with intraperitoneal administration of PTX, intravenous administration of oxaliplatin, and oral S-1 were done. Then three courses with intraperitoneal and intravenous administration of PTX and oral S-1 were done. Oxaliplatin was administered intravenously at a dose of 100 mg/m^2^ on day 1, and PTX was administered intravenously at a dose of 50 mg/m^2^ on day 1 and intraperitoneally via the access port at a dose of 20 mg/m^2^ on days 1 and 8, respectively. PTX was diluted in 1 l normal saline over 1 h. S-1 was administered orally twice daily at a dose of 80 mg/m^2^ per day for 14 consecutive days, followed by 7 days without treatment. These chemotherapy in clinical studies were approved by the institutional review board of The University of Tokyo. Repeat laparoscopy showed disappearance of the peritoneal carcinomatosis (Fig. 2a–d), after which she underwent total gastrectomy with lymphadenectomy. Pathological examination revealed no viable cancer cells remaining in the resected specimen (Fig. 3). The patient received a total of 38 courses of chemotherapy with intraperitoneal and intravenous administration of PTX and oral S-1 after the gastrectomy, without apparent metastasis or recurrence.

Two years and 6 months after the surgery, she experienced lumbago, with increases in the carcinoembryonic antigen (CEA), carbohydrate antigen (CA) 19-9, and CA125 levels observed. Computed tomography showed bone metastases at the thoracic to lumbar vertebrae. A decreased platelet count was noted, indicating DIC, and the patient was hence readmitted to our hospital.

**Fig. 4:**
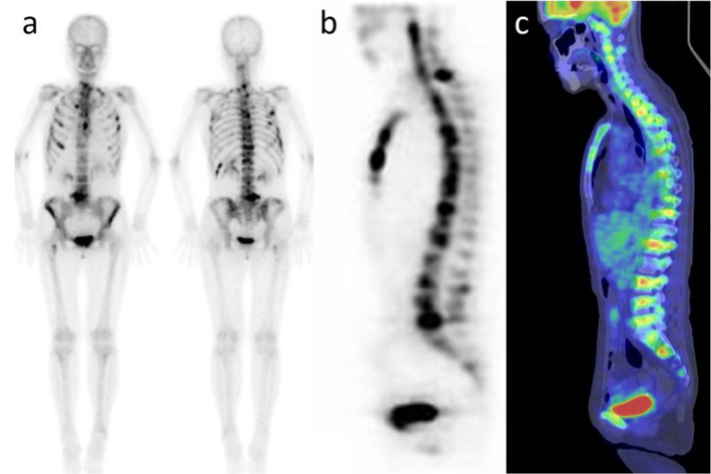
**a**, **b** Bone scintigraphy and **c** fluorodeoxyglucose-positron emission tomography of the patients. Multiple bone metastases to the thoracic to lumbar vertebrae, costae, sternum, iliac bone, scapulae, and femora are shown, suggestive of disseminated carcinomatosis of the bone marrow

At the time of admission, percutaneous bleeding was observed in her forearm. Blood test showed hemoglobin, 9.9 g/dl; platelet count, 72,000/mm^3^; alkaline phosphatase, 1969 IU/l; prothrombin time international normalized ratio, 1.17; fibrinogen, 346 mg/dl; and fibrinogen degradation products, 201.1 μg/ml. The patient was diagnosed as DIC, and treatment with gabexate mesilate was initiated. The tumor marker levels, including CEA (426.3 ng/ml) and CA19-9 (2413 IU/ml), were also further increased. Bone scintigraphy and fluorodeoxyglucose-positron emission tomography showed multiple bone metastatic lesions in the thoracic to lumbar vertebrae, costae, sternum, iliac bone, scapulae, and femora (Fig. 4a–c). DCBM from gastric cancer was clinically diagnosed, and chemotherapy was immediately commenced with a regimen of weekly irinotecan (CPT-11). On day 1, intravenous CPT-11 (100 mg/m^2^, 80 % dose) was administered, and the regimen was repeated on a weekly basis. After three courses of chemotherapy, she recovered from DIC with relief of the lumbago. The same regimen was repeated in an outpatient setting for 5 months. However, although the patient did not show any signs of peritoneal recurrence, the tumor markers increased and DIC developed again, and the patient died of DCBM 3 years and 5 months after the introduction of intraperitoneal chemotherapy.

## Discussion

In the present case, gastric cancer metastasized to the bone marrow and caused DCBM associated with DIC 3 years after intraperitoneal chemotherapy against peritoneal carcinomatosis of gastric cancer. DCBM is uncommon in patients with gastric cancer; Kim et al. reported that only 2.4 % of metastatic, unresectable, or recurrent gastric cancers had confirmed bone marrow metastasis [5]. And DCBM from solid tumors was closely associated with DIC [6]. Jarcho et al. reported an association between multiple bone metastases and DIC in diffusely infiltrative gastric cancer as early as 1936 [7], and Brain et al. reported a close association between mucin-forming cancer and DIC and/or microangiopathic hemolytic anemia [8]. Later, Pasquini et al. examined bone metastases from solid tumors with hematological disorders, and the prognosis is very poor [9]. This condition is called DCBM [5, 6].

In patients with DCBM and DIC from solid tumors, gastric cancer accounts for the majority of cases [9]. DCBM is associated with various symptoms, including bone pain, bleeding tendencies, anemia, general weakness, and elevated levels of serum alkaline phosphatase [5, 6, 10]. Macroscopic Borrmann types III and IV, histologically poorly differentiated adenocarcinoma, younger age, highly advanced tumors, and primary tumors in the upper part of the stomach are risk factors for gastric cancer resulting in DCBM [5, 6, 10]. Of note, many cases (66.7 %) recur over 5 years following the initial surgery, with recurrence at the bone marrow more than 10 years as reported in some case [11, 12], indicating the presence of “tumor dormancy,” a long latent phase of tumor progression [13]. The present case matched most of these risk factors and presented all major symptoms described in the literature in the 2 months after presenting with lumbago.

DCBM is associated with a poor prognosis, with some patients dying within a few weeks as a result of bleeding or organ insufficiency caused by DIC [5, 6, 10]. Park et al. examined 203 cases of bone marrow metastasis originating from gastric cancer. They found that the median survival time was 103 days, with complicated DIC being associated with a significantly poorer prognosis, while patients receiving chemotherapy had a better prognosis (175 days). On the other hand, the median survival time of the patients who did not receive chemotherapy was only 43 days [14], indicating that aggressive chemotherapy is effective for DCBM [10–12]. In this case, we changed the regimen to weekly CPT-11 because the patient had already received other key drugs for gastric cancer, including S-1, cisplatin, and PTX. The patient overcame DIC once and survived for 6 months after the diagnosis of DCBM.

In our previous clinical studies, we treated peritoneal carcinomatosis of gastric cancer using intraperitoneal chemotherapy with PTX. In this intraperitoneal chemotherapy, PTX is repeatedly administered into the intraperitoneal space via a subcutaneously placed intraperitoneal access port. This combination chemotherapy is effective for peritoneal metastases and appears to produce a marked elongation of survival in these patients [1–3]. Moreover, peritoneal carcinomatosis often disappears macroscopically during the course of intraperitoneal chemotherapy. From August 2005 to April 2012, 158 cases of gastric cancer with peritoneal carcinomatosis underwent intraperitoneal chemotherapy with PTX in our department. Recurrence in the bone was found in 15 cases (9.5 %), with multiple bones implicated in 4 cases (2.5 %); of these, 3 cases (1.8 %) were clinically suspected as DCBM with DIC. Two of these three cases (with the exception of the present case) were treated with best supportive care and died within 1 month. The higher rate of DCBM experienced in our department compared with that in previous reports may be due to the strong suppression of peritoneal carcinomatosis by intraperitoneal administration of PTX.

## Conclusions

We here experienced a patient of gastric cancer with peritoneal carcinomatosis who developed DCBM 3 years after initiation of intraperitoneal chemotherapy with PTX. Since intraperitoneal PTX administration can strongly suppress peritoneal carcinomatosis of gastric cancer, careful attention should be paid not only to peritoneal recurrence but also for rare site metastases, such as bone marrow metastases, during the course of intraperitoneal chemotherapy.

### Consent

Written informed consent was obtained from the patient for publication of this case report and any accompanying images. A copy of the written consent is available for review by the Editor-in-Chief of this journal.

## Abbreviations

CA: carbohydrate antigen
CEA: carcinoembryonic antigen
CPT-11: irinotecan
DCBM: disseminated carcinomatosis of the bone marrow
DIC: disseminated intravascular coagulation
PTX: paclitaxel
